# MicroRNAs: Promising New Antiangiogenic Targets in Cancer

**DOI:** 10.1155/2014/878450

**Published:** 2014-08-14

**Authors:** Sandra Gallach, Silvia Calabuig-Fariñas, Eloisa Jantus-Lewintre, Carlos Camps

**Affiliations:** ^1^Molecular Oncology Laboratory, General University Hospital Research Fundation, Avda Tres Cruces 2, 46014 Valencia, Spain; ^2^Department of Biotechnology, Universitat Politècnica de València, Camino de Vera s/n, 46022 Valencia, Spain; ^3^Medical Oncology Department, General University Hospital, Avda Tres Cruces 2, 46014 Valencia, Spain; ^4^Department of Medicine, Universitat de València, Avda Blasco Ibañez 15, 46010 Valencia, Spain

## Abstract

MicroRNAs are one class of small, endogenous, non-coding RNAs that are approximately 22 nucleotides in length; they are very numerous, have been phylogenetically conserved, and involved in biological processes such as development, differentiation, cell proliferation, and apoptosis. MicroRNAs contribute to modulating the expression levels of specific proteins based on sequence complementarity with their target mRNA molecules and so they play a key role in both health and disease. Angiogenesis is the process of new blood vessel formation from preexisting ones, which is particularly relevant to cancer and its progression. Over the last few years, microRNAs have emerged as critical regulators of signalling pathways in multiple cell types including endothelial and perivascular cells. This review summarises the role of miRNAs in tumour angiogenesis and their potential implications as therapeutic targets in cancer.

## 1. Introduction

MicroRNAs (miRNAs) were initially discovered in 1993 by Lee et al. while they were studying the* lin-4* gene. They showed that* lin-4* encodes a small RNA with antisense complementarity to the* lin-14* gene which resulted in reduced lin-14 protein expression and thus disrupted the regulation of developmental timing of the nematode* Caenorhabditis elegans* [1]. miRNAs were subsequently shown to inhibit their target genes through specific sequences which are complementary to the target messenger RNA (mRNA). This discovery resulted in a paradigm shift in our understanding of gene regulation because miRNAs are now known to repress thousands of target genes and to coordinate many physiological processes including, but not limited to, development, differentiation, cell proliferation, and apoptosis [2–4]. The aberrant expression or alteration of miRNAs also contributes to a range of human pathologies, including cancer [5–7].

## 2. MicroRNAs: Definition, Biogenesis, and Function

miRNAs are one class of small noncoding RNAs that are approximately 18–25 nucleotides in length; they are evolutionary conserved single-stranded RNA molecules which are involved in the specific regulation of gene expression in eukaryotes [8–10]; thousands have been identified in a wide variety of species. They can increase or decrease protein expression by binding to the 3′-untranslated region (UTR) or to other regions (e.g., the 5′-UTR, coding sequences) of target mRNA transcripts [11] and thus play a central role in gene regulation in both health and disease. miRNA genes are located in inter- or intragenic regions of protein-coding gene introns and/or exons and are transcribed from DNA but not translated into proteins; they can exist individually or form clusters (reviewed in [12]).

miRNA biogenesis starts with transcription from a miRNA gene by RNA polymerase II (pol II), generating a primary transcript RNA (pri-miRNA) which is up to several kilobases in length [13] and which can be distinguished by the presence of an imperfect double stranded (ds) RNA region known as the stem-loop structure. This structure is recognised by the nuclear RNase III endonuclease Drosha and its cofactor DGCR8 (DiGeorge syndrome critical region 8) which together with other proteins form a complex known as the microprocessor complex that cleaves the pri-miRNA and releases an approximately 60–70 nt long precursor miRNA (pre-miRNA) [14–17]. The pre-miRNA is exported from the nucleus to the cytoplasm via the exportin-5 protein (RAN-GTP-dependent transporter) [18–20] and once the complex is in the cytoplasm, Dicer (RNase III endonuclease), with the help of TRBP (the human immunodeficiency virus transactivating) and AGO2 (argonaute 2), generates the final mature 18–25 nt ds miRNA, miRNA:miRNA* (the complementary miRNA strand, referred to as miRNA*) [21, 22]. The mature miRNA loses one of its strands and the remaining one is loaded onto an argonaute-containing RNA-induced silencing complex (RISC) to form a miRISC which mediates protein inhibition [23, 24].

**Figure 1:**
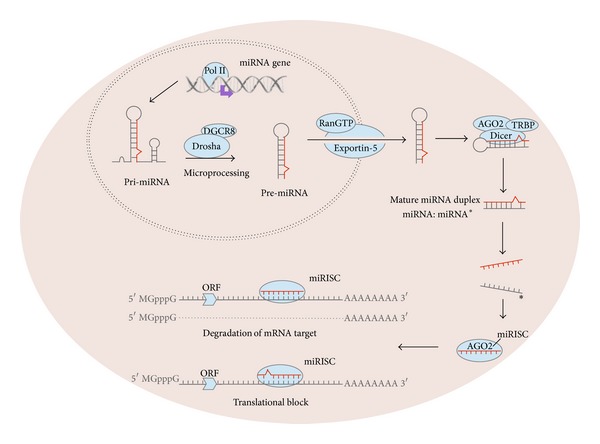
miRNA biogenesis: miRNA gene transcription generates primary miRNA (pri-miRNA) in the nucleus which is then cleaved by the microprocessor complex (Drosha and DGCR8), liberating pre-miRNA which is exported from the nucleus to the cytoplasm by exportin-5. Pre-miRNA is finally processed by Dicer and TRBP to obtain a mature miRNA with the capacity to bind to target mRNAs. AGO2: argonaute 2, DGCR8: DiGeorge syndrome critical region 8, miRISC: miRNA bound to RNA-induced silencing complex, ORF: origin replication frame, Pol II: polymerase II, and TRBP: the human immunodeficiency virus transactivating.

Once a miRNA binds to its target gene, two mechanisms of action are known: (i) mRNA degradation and (ii) translational mRNA inhibition without degradation, the latter of which occurs in animals, including mammals [25]. In the first of these mechanisms the binding is completely complementary between the miRNA and mRNA sequences whereas in the second one, where the bound mRNA remains untranslated, the binding is not completely complementary, resulting in reduced target gene expression (Figure 1). Another important characteristic of miRNAs is that one single miRNA has the potential to regulate many target genes while any one gene can be targeted by multiple miRNAs, meaning that miRNAome-mRNAome interaction can be a complicated network.

Some data in humans have shown that about 30–50% of genes coding for proteins are controlled by miRNAs [26]; therefore, any signalling pathway or cellular mechanism could potentially be governed by them. The causes of miRNA dysregulation in cancer can result from various mechanisms including (reviewed in [27]) the deletion or amplification of miRNA-coding chromosomal regions [6, 28–30], mutations in the miRNA or the target site sequence of its respective gene(s) [31–34], epigenetic silencing of miRNA promoters [35–38], or the dysregulation of proteins upstream of the miRNA pathway such as cellular signalling and transcription factors [39–45]. Hence, the ability of miRNAs to simultaneously regulate several genes makes them a very attractive study target, especially, given that many tumour cell types have altered miRNA expression patterns. In particular, recent work has provided support for the idea that noncoding RNAs, and in particular miRNAs, may play important roles in physiological and pathological angiogenesis.

## 3. Tumour Angiogenesis

Tumour angiogenesis is the process by which new blood vessels form in neoplasms; it starts in the early stages of disease and is a crucial step in the growth and spread of tumours. Without forming new blood vessels tumours cannot grow beyond a certain size due to the lack of oxygen and other essential nutrients [46]. Neovascularization has a dual effect on the tumour: firstly it supplies nutrients, oxygen, and growth factors that stimulate tumour cell growth [47]. Secondly, in combination with lymphangiogenesis, it is a prerequisite for metastasis as it provides a site of entry into the circulation allowing shed tumour cells to travel through the bloodstream to reach remote organs [48]. This pathological angiogenesis is characterized by uncontrolled growth and disordered vasculature and appears when there is an imbalance between pro- and antiangiogenic factors [49].

In order to initiate the neovascularization, tumour cells may overexpress one or more angiogenic inducers, mobilise proangiogenic proteins from the extracellular matrix, or attract host cells such as macrophages which produce their own angiogenic proteins [50].

The activation of angiogenesis starts when preexisting vessels become permeabilised in response to stimulating factors such as VEGF (vascular endothelial growth factor), PLGF (placental growth factor), or ANG-1 (angiopoietin-1). The basement membrane and extracellular matrix (ECM) are locally degraded by extracellular matrix metalloproteinases (MMPs) allowing the underlying endothelial cells (ECs), which are attracted by the angiogenic stimulus produced by the tumour cells and the microenvironment, to migrate into the perivascular space [51]. In tumour vasculature, the pericyte coating is decreased or is inadequate, leading to the formation of fenestrations and/or transcellular holes; these incomplete basal membranes and the fact that tumour blood vessel walls can also be formed by both endothelial and tumour cells lead to the formation of vessels with irregular diameters and structural abnormalities [51, 52].

In summary, angiogenesis is regarded as an essential step in cancer development which promotes tumour progression and metastasis by providing an entry site into the circulation [53]. Angiogenesis has become the focus of intense study in recent years, for example, in the development of antiangiogenesis pharmacological agents as attractive antitumor targets [54, 55]. In addition, the response of the vascular endothelium to angiogenic stimuli is modulated by certain miRNAs which can be either proangiogenic or antiangiogenic. For this reason, the study of miRNAs and angiogenesis is likely to improve our understanding of the process of carcinogenesis and may lead to the identification of new therapeutic targets for cancer treatment.

## 4. Role of MicroRNAs in the Regulation of Angiogenesis

### 4.1. Enzymes Involved in miRNA Biogenesis

One approach to studying the biological relevance of miRNAs is by silencing their functions by mutating or disrupting Dicer, a critical enzyme involved in miRNA maturation [22]. Functional loss of Dicer results in profound vascular developmental abnormalities in both zebrafish and mice [56, 57], but the first evidence that miRNAs were involved in the regulation of angiogenesis during vascular development came from investigating mice with hypomorphic Dicer expression; these mice had a retarded phenotype and died as embryos between days 12.5 and 14.5 because Dicer is specifically required for the formation/maintenance of blood vessels in embryos and yolk sacs [58]. Furthermore, these mutant embryos also had altered* Vegf*,* Flt1*,* Kdr* (kinase insert domain receptor), and* Tie2* expression indicating that Dicer probably exerts its function because it is involved in the biogenesis of miRNAs that regulate the expression levels of these critical proangiogenic factors in mice [58]. Similarly, generation of mutant embryos disrupts Dicer in zebrafish and results in pericardial oedema and vascular defects [59]. Moreover, genetic silencing of Dicer and/or Drosha in HUVECs reduces EC proliferation, migration, capillary sprouting, and tube forming activity* in vitro* and, in the case of Dicer (but not Drosha), reduces angiogenesis* in vivo* [60, 61]. This difference in the effects of Dicer and Drosha might be due to a recently described alternative Drosha processing pathway which is miRNA-independent [62]. Another new study in bone marrow mice endothelial progenitor cells (EPCs) also showed that conditional ablation of Dicer led to the inhibition of angiogenesis and impaired tumour growth, demonstrating that functional Dicer is also necessary for bone marrow-mediated tumour angiogenesis [63]. Together, these studies reveal that Dicer and Drosha are prerequisite enzymes in miRNA processing and demonstrate the essential role of miRNAs in angiogenesis.

### 4.2. MicroRNAs and Endothelial Cells

Different cell types contribute to tumour neovascularization; among them, the endothelial and perivascular cells are generally acknowledged to play a central role in the angiogenesis process. miR-126 was suggested to be an endothelium specific miRNA, which modulates the endothelial phenotype* in vitro *and blood vessel integrity* in vivo,* respectively [64]. It is encoded by intron 7 of the* EGFL7* (EGF-like domain 7) gene, which encodes an EC-specific secreted peptide that acts as a chemoattractant and smooth muscle cell migration inhibitor [65–67]; both miR-126 and* Egfl7* have a similar EC expression pattern [68]. In concordance, it has been demonstrated that this miRNA is enriched in tissues with a high vascular component such as the lung and heart [69, 70]. miR-126 promotes angiogenesis in response to VEGF and bFGF (basic fibroblast growth factor) through negative suppression regulators in signal transduction pathways [64, 68, 71]. Furthermore, miR-126 has been shown to be enriched in Flk-1 (kinase insert domain receptor; a type III VEGF receptor tyrosine kinase) positive cells derived from mouse embryonic bodies. miR-126 also directly regulates the vascular process by targeting* SPRED-1* (sprouty-related,* EVH1* domain containing 1),* VCAM1* (vascular cell adhesion molecule 1), and* PIK3R2* (phosphoinositide-3-kinase, regulatory subunit 2, also known as p85-*β*) resulting in posttranscriptional repression in HeLa cells [64]. miR-126 loss-of-function studies in both mice and zebrafish highlighted its importance in developmental and pathological angiogenesis affecting the EC function* in vivo* [64]. Targeted deletion of miR-126 in mice causes leaky vessels, hemorrhaging, and partial embryonic lethality, due to a loss of vascular integrity and defects in EC proliferation, migration, and angiogenesis; these vascular abnormalities are similar to those caused by diminished angiogenic growth factor signalling (e.g., by VEGF or FGF). miR-126 enhances MAP kinase signalling in response to VEGF and FGF and, in its absence, angiogenic growth factor signalling is reduced. This process may be regulated by* Vegf *suppression mediated by* Spred-1*, considering that it is a negative regulator of the RAS/MAP kinase pathway; therefore, miR-126 promotes blood vessel formation by repressing SPRED-1 expression [68]. Another finding was that miR-126 deletion inhibits VEGF-dependent AKT and ERK signalling derepressing the p85*β* subunit of* Pi3-kinase* and of* Spred-1*, respectively [71]. Finally, Png and colleagues reported that miR-126 regulates EC recruitment to metastatic breast cancer cells* in vitro* and* in vivo* [72]. According to these data, it seems that miR-126 contributes to the EC recruitment in physiological as well as in pathological conditions and might be a promising antiangiogenic target.

Other miRNAs have been found to regulate the angiogenic process by exerting an antiangiogenic function. Among these miR-221 and miR-222 are highly conserved miRNAs which are transcribed from a pri-miRNA located on the human X chromosome. These miRNAs are negative regulators of angiogenesis [73], have a proliferative effect on cancer cells [74], and are also expressed by growth factor-stimulated or quiescent ECs [11]; indeed, microarray data indicate that these are the most abundantly expressed miRNAs in HUVECs [73]. This latter study showed that these two miRNAs inhibit stem cell factor (SCF) by decreasing the abundance of c-KIT (tyrosine kinase receptor for SCF), thus blocking EC migration, proliferation, and angiogenesis* in vitro*. Their antiangiogenic activity was further demonstrated by their interaction with the c-KIT 3′-UTR in ECs [75] and this group also showed that these two miRNAs regulate endothelial nitric oxide synthase (eNOS) in ECs by silencing Dicer [61]. NO is synthesized by eNOS and is necessary for EC survival, migration, and angiogenesis [76]. However, binding sites for these miRNAs were not found in the eNOS 3′-UTR, suggesting that miR-221/222 are likely to indirectly regulate eNOS protein production. These miRNAs can also specifically promote cancer cell proliferation by regulating the* p27* (Kip1) tumour suppressor gene [74], indicating that the regulation of proliferation by miR-221/222 is cell-type specific. More recent studies have shown that these miRNAs control different target genes: miR-222 is involved in inflammation mediated by vascular growth factors [77], whereas miR-221 is required for vascular remodelling [78]. Similarly, a study performed in a murine model of liver tumorigenesis showed that miR-221 but not miR-222 accelerated tumour growth [79].

Similar to miR-221/222, the polycistronic miR-17-92 gene cluster (Cl3orf25), located on human chromosome 13q31.3, which encodes six mature miRNAs, miR-17, miR-18a, miR-19a, miR-20a, miR-19b, and miR-92a [80], is also highly expressed in ECs [81]. This cluster is amplified in several types of lymphoma and solid tumours [45, 82] and regulates vascular integrity and angiogenesis, promoting tumour neovascularization* in vivo* by downregulating antiangiogenic* THBS1* (thrombospondin 1) [83]. A recent study showed that while miR-17, miR-18a, and miR-19a expression were enhanced and miR-92a expression was reduced during EC differentiation, inhibiting these miRNAs did not affect EC differentiation [84]. Although the cluster is highly upregulated in several human tumour types, only miR-18a and miR-19a have a proangiogenic function during tumour angiogenesis [71, 85, 86]. In contrast, an antiangiogenic role for miR-17-92 cluster members has also been reported in two different studies in cultured ECs [86, 87]: the first reported an antiangiogenic role for miR-92a in ECs, where injection of miR-92a antagomirs (small synthetic RNAs that are perfectly complementary to the specific miRNA to inhibit its function) into mice promoted neovascularization in ischemic limbs. This antiangiogenic function was mediated by ITGA5 (integrin *α*5 subunit) repression and also indirectly suppressed eNOS production [87]. The second showed that overexpression of miR-17, miR-18a, miR-19a, miR-20a, and miR-19b inhibited EC sprouting, network formation, and cell migration, which was reversed when they were silenced [86]. However, the combined antagomir inhibition of miR-17 and miR-20a* in vivo* enhanced vessel invasion into subcutaneous tissue, although it did not enhance tumour angiogenesis; relevant targets for this miR-17 antiangiogenic activity include the cell cycle inhibitor* p21*, the S1P receptor EDG1, and the protein kinase JAK-1 [86]. A new study has shown that miR-17-3p controls the angiogenic process in HUVECs* in vitro* in a cell-autonomous manner by modulating the FLK-1 (VEGFR-2) expression implicated in the pleiotropic effects of angiogenesis. miR-17-3p negatively regulates FLK-1-mediated angiogenesis in ECs by rapidly downregulating expression via a 21 bp fragment from the* FLK-1* 3′-UTR [88]. Thus both the pro- and antiangiogenic properties of the miR-17-92 cluster seem to be related to the cellular microenvironment.

Inhibition of Dicer and Drosha by siRNAs reduces let-7f and miR-27b expression in ECs* in vitro*, and inhibitors for both miRNAs contribute to the reduction of* in vitro* angiogenesis and sprout formation, suggesting that let-7f and miR-27b promote angiogenesis by targeting antiangiogenic genes such as* THBS1* (using* in silico* analysis of predicted targets), although these targets have not yet been characterised [60, 75]. Furthermore, miR-214 overexpression in ECs significantly inhibited tubular sprouting, and, similarly, knockdown of the quaking protein (a direct miR-214 target which is critical for vascular development) reduced proangiogenic growth factor expression and EC sprouting; moreover, miR-214 upregulation decreased the secretion of proangiogenic growth factors, including VEGF, which was reversed by inhibiting it [89].

Finally, Fang et al. reported that miR-93, a miRNA from the miR-106B-25 cluster and a paralog of the miR-17-92 cluster, has both pro- and antiangiogenic properties. It enhanced EC activities, including cell spreading and tube formation in a human breast carcinoma cell line by targeting the* LATS2* gene (large tumour suppressor kinase 2), whereas it was found to be upregulated in human breast carcinoma tissues [90].

**Figure 2:**
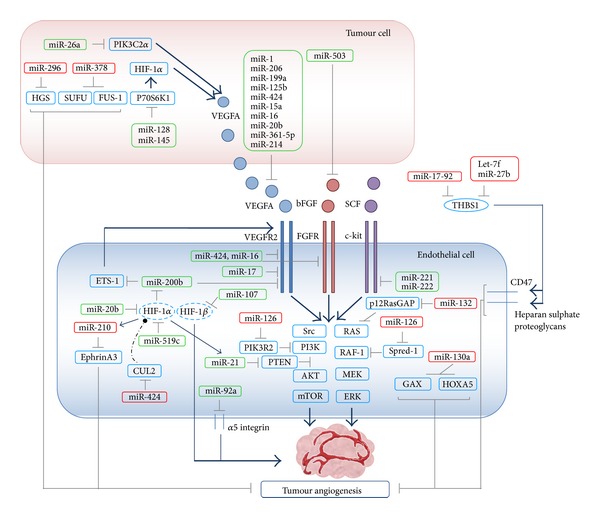
miRNAs involved in angiogenic process. Angiogenesis regulation conducted by different miRNAs is based on a complex network and is summarized in this figure. Red boxes indicate proangiogenic miRNA, green boxes indicate antiangiogenic miRNAs, and dashed circles indicate genes involved in molecular pathway taking place in both tumour and endothelial cells. Grey lines represent inhibitory processes while the blue lines with arrows represent activation processes and the dashed black line represents the ubiquitin-mediated degradation HGS (hepatocyte growth factor-regulated tyrosine kinase substrate), SUFU (suppressor of fused), FUS-1 (FUS RNA binding protein), PIK3C2*α* (phosphoinositide-3-kinase class 2*α*), THBS1 (thrombospondin-1), HIF-1*α* (hypoxia-inducible factor 1 alpha), HIF-1*β* (hypoxia-inducible factor-1 beta), VEGF (vascular endothelial growth factor), bFGF (basic fibroblast growth factor), Spred-1 (sprouty-related, EVH1 domain containing 1), PIK3R2 (phosphoinositide-3-kinase, regulatory subunit 2), SCF (stem cell factor), c-KIT (tyrosine-protein kinase kit), VEGFR-2 (vascular endothelial growth factor receptor 2), ERK (extracellular signal-regulated kinase), AKT (v-akt murine thymoma viral oncogene homolog 1), PTEN (phosphatase and tensin homolog), Ets-1 (avian erythroblastosis virus E26 (v-ets) oncogene homolog-1), fibroblast growth factor receptor-1 (FGFR-1), GAX (growth arrest homeobox) and HOXA5 (homeobox A5), RAS (v-ki-ras2 kirsten rat sarcoma viral oncogene homolog), RAF-1 (v-raf-1 murine leukemia viral oncogene homolog 1), Cul2 (Cullin 2), mTOR (mechanistic target of rapamycin serine/threonine kinase), and Src (v-src avian sarcoma (Schmidt-Ruppin A-2) viral oncogene homolog).

The most important mechanisms and functions involved in EC regulation by miRNAs described above are summarized in Figure 2 and Table 1.

### 4.3. miRNAs and Hypoxia

Hypoxia, a key driver of angiogenesis, works primarily by inducing angiogenic factors via the HIF-1*α* (hypoxia-inducible factor-1 alpha) pathway. Hypoxia occurs during tumour development, and several hypoxia-regulated miRNAs have been identified in cancer cells, as detailed below.

miR-210 is the only miRNA so far identified which strongly responds to hypoxic stress in virtually all experimental systems* in vivo* and* in vitro* and in both normal and tumour cells under physiological hypoxic conditions [91]. Hypoxia in tumours is closely related with angiogenesis [92] and several proangiogenic factors are overexpressed in tumours as a response to a hypoxic microenvironment [93], VEGF being the best example [94, 95]. miR-210 and* VEGF* expression were closely correlated in breast cancer patients [96], showing a possible role for miR-210 in tumour angiogenesis. In support of this, two independent studies demonstrated that upregulation of miR-210 in normoxic HUVECs induced the formation of capillary-like structures and VEGF-dependent EC migration, while inhibiting it antagonised these processes [97, 98]. Furthermore, miR-210 induces angiogenesis in part repressing endothelial ligand ephrin-A3, which is an antiangiogenic factor [97]. In another study HUVECs cultured with exosomes derived from mouse breast cancer 4T1 cells which were transfected with miR-210 had significantly increased migration and capillary formation [99]. Taken together, this data suggests that miR-210 may be one of the angiogenesis-promoting factors released by tumour cells, therefore explaining the increased quantities of miR-210 found in the circulation of cancer patients [100, 101].

Two studies performed in four different murine tumour cell lines as well as the MCE-7 breast cancer cell line showed that miR-20b regulates angiogenesis by targeting VEGF and HIF-1*α* [102, 103].While repression of miR-20b enhanced HIF-1*α* and VEGF protein levels in normoxic conditions, hypoxic conditions increased miR-20b levels and decreased HIF-1*α* and VEGF levels. Overexpression of HIF-1*α* also downregulated miR-20b expression in normoxic tumour cells, whereas HIF-1*α* repression in hypoxic tumour cells caused miR-20b to increase. It is thought that this might be a novel molecular regulation mechanism through which miR-20b regulates HIF-1*α* and VEGF but which is also self-regulated by HIF-1*α* so that tumour cells continuously adapt to different oxygen concentrations [103]. In support of this idea Cascio et al. used hypoxia-mimicking conditions (CoCl_2_ exposure) to demonstrate that VEGF expression in breast cancer cells is mediated by HIF-1*α* and STAT3 in a miR-20b-dependent manner. miR-20b decreased VEGF protein levels after CoCl_2_ treatment, and* VEGF* mRNA downregulation by miR-20b was associated with reduced levels of nuclear HIF-1*α* and STAT3; STAT3 was also necessary for CoCl_2_-mediated HIF1*α* nuclear accumulation and its recruitment to the* VEGF* promoter [102].

Additionally, miR-21 has been identified as one of the most important miRNAs associated with tumour growth and metastasis. Lei et al. confirmed that its overexpression in DU145 cells increases both HIF-1*α* and VEGF expression to promote tumour angiogenesis and that, similar to previous findings [103], HIF-1*α* (itself a key downstream miR-21 target) downregulation negated miR-21-induced tumour angiogenesis. miR-21 activates AKT and ERK 1/2 (extracellular signal-regulated kinases) by targeting PTEN (phosphatase and tensin homolog) which elevates HIF-1*α* and VEGF expression [104]. Interestingly, miR-21 is only upregulated by hypoxia in AKT2-expressing cells, and AKT2 confers greater resistance to hypoxia than AKT1 or AKT3. When miR-21 is upregulated in hypoxic conditions AKT2 downregulates PTEN which then activates the other two Akt isoforms. In addition, miR-21 also downregulates PDCD4 (programmed cell death 4) and sprouty 1 (Spry1) which, together with PTEN downregulation, confers resistance to hypoxia [105]. This group also confirmed the involvement of the AKT2/miR-21 pathway in angiogenesis* in vivo* in hypoxic human ovarian carcinoma cells and in the MMTV-PyMT (mouse mammary tumour virus-polyoma-middle T) breast cancer metastasis model. Taken together, these data indicate that a novel AKT2-dependent pathway is activated by hypoxia and that this promotes tumour resistance by inducing miR-21.

The miR-200 family plays a crucial role in epithelial-to-mesenchymal transition by controlling cell migration and polarity [106–108]. Delivery of miR-200b mimic into HMECs (human microvascular endothelial cells) suppressed the angiogenic response, whereas miR-200b-depleted HMECs exhibited elevated angiogenesis* in vitro*, as evidenced by Matrigel tube formation and cell migration assays [109]. Using different technologies, this group showed that (i) ETS-1 (avian erythroblastosis virus E26 (v-ets) oncogene homolog-1), an essential angiogenesis-related transcription factor, is a direct miR-200b target, (ii) some* ETS-1*-associated genes such as* MMP-11* and* VEGFR-2* were downregulated by miR-200b, and (iii) hypoxia and HIF-1*α* stabilisation inhibited miR-200b expression, increasing* ETS-1* expression. As miR-200b becomes downregulated in a hypoxic environment its expression is derepressed and angiogenesis is promoted [109]. A recent study on the A549 and HUVEC cell lines demonstrated that miR-200c regulates* VEGFR-2* expression, increasing cancer cell radiosensitivity by targeting the VEGF-VEGFR-2 pathway. Ectopic miR-200c expression in HUVECs significantly impaired angiogenesis, tubulogenesis, and migration, whereas miR-200c suppression increased tube formation and migration by about 30% [110].

In addition to the key miRNAs discussed above, miR-107, miR-519c, and miR-424 have also been implicated in hypoxia-induced angiogenesis. Yamakuchi et al. showed that miR-107 decreases hypoxic signalling in human colon cancer cells by inhibiting* HIF-1β* and that it is transcriptionally regulated by p53; in addition, miR-107 overexpression in mouse tumour cells repressed tumour angiogenesis, growth, and VEGF expression [111]. Cha et al. identified miR-519c as another important hypoxia-independent regulator which directly binds the* HIF-1*α 3′UTR and thus causes a reduction in tumour angiogenesis. Overexpression of this miRNA significantly decreased HIF-1*α* protein levels and reduced HUVEC tubulogenesis, whereas its inhibition by antagomirs had the opposite effect [112]. miR-424, which is increased in hypoxic ECs and during vascular remodelling* in vivo*, is thought to play an important role in postischemic vascular remodelling and angiogenesis. It inhibits CUL2 (Cullin 2) expression by targeting its 3′-UTR, stabilising HIF-1*α*, which then transcriptionally activates VEGF; similarly, EC transfection with miR-424 increases both HIF-1*α* and HIF-2*α* expression and increases proliferation and migration, presumably through the same VEGF-mediated mechanism [113].

In summary, many different studies have identified the functional targets and pathways involved in miRNA-mediated regulation of hypoxia (Figure 2 and Table 1), providing a rationale for a new therapeutic approach to suppressing hypoxia-induced tumour angiogenesis.

### 4.4. miRNAs and the VEGF Pathway

The first miRNAs which were directly associated with tumour biology by their downregulation or deletion were miR-15a and miR-16; expression of these miRNAs is reduced in response to hypoxia which increases VEGF expression [114]. These miRNAs also induce apoptosis in leukaemia cells by inhibiting* BCL-2* (an antiapoptotic protein) and blocking cell cycle progression, making them attractive antitumour targets which could be used to block tumour cell survival, proliferation, and VEGF-mediated angiogenesis [115]. miR-15a and miR-16 are significantly underexpressed in primary multiple myeloma (MM) cells as well as MM cell lines and their expression inversely correlates with VEGF in both human MM cell lines and normal plasma cells [116]. Moreover, miR-16 and another miRNA, miR-424, decrease VEGF, VEGFR-2, and FGFR-1 (fibroblast growth factor receptor-1) expression (all of which have been validated as miR-16 and miR-424 targets in ECs using mimetic microRNAs) and hence play an important role in regulating the cell-intrinsic angiogenic activity of ECs by increasing VEGF and bFGF signalling [117]. Dejean et al. showed that miR-16 directly interacts with* VEGF *mRNA at the 3′-UTR and that ALK expression leads to miR-16 downregulation, thus increasing VEGF levels. This was further supported by experiments in TPM3-ALK (conditional onco-ALK) MEF cells which showed that both ALK and HIF1*α* expression are a prerequisite for miR-16 downregulation; in agreement with these findings, increased miR-16 expression* in vivo* reduced angiogenesis and tumour growth [118].

miR-378 is another important angiogenic regulator. When this miRNAs is overexpressed in cancer cell lines,* SUFU* (suppressor of fused) and* FUS-1* (FUS RNA binding protein), two tumour suppressor genes, are downregulated, and as a consequence there is an increase in the levels of VEGF, thus increasing cell survival and reducing cell death [119].

Similarly, increased miR-296 expression activates angiogenesis in cultured ECs due to the suppression of HGS (hepatocyte growth factor-regulated tyrosine kinase substrate) which mediates VEGFR-2 and PDGFR-*β* (platelet derived growth factor receptor beta) degradation, whereas miR-296 inhibition reduces angiogenesis in tumour xenografts [120]. In contrast, He et al. identified another two miRNAs, miR-199a and miR-125b, which were downregulated in ovarian cancer tissues and cell lines, and overexpression of these miRNAs inhibits tumour-induced angiogenesis and is associated with a decrease in* VEGF* mRNA and protein expression [121]. A different miRNA, miR-361-5p, represses a miRNA recognition element located in a conserved downstream region of the* VEGFA* 3′-UTR and is inversely correlated with VEGFA expression in human squamous cell carcinoma (SCC) cells and in healthy skin, indicating that it may play a role in cancers [122].

Two studies in zebrafish have demonstrated that miR-1, miR-206, and miR-10 govern angiogenesis by targeting* vegf* [123, 124]. They negatively regulate developmental angiogenesis by controlling VegfA in muscle and thus angiogenic signalling to the endothelium. Interestingly, reducing the levels of VegfAa but not VegfAb rescued the increase in angiogenesis previously observed when miR-1/206 were knocked down [124]. miR-10 repression led to premature truncation of intersegmental vessel growth in the trunk of zebrafish larvae, and its overexpression promoted angiogenesis in both zebrafish and cultured HUVECs. miR-10 acts by directly regulating FLT1 (a cell-surface receptor that binds VEGF) and its soluble splice variant SFLT1. Its downregulation in zebrafish and HUVECs increases FLT1/SFLT1 protein levels, which binds VEGF with higher affinity than VEGFR-2 and therefore negatively regulates VEGFR-2 signalling pathway [123]. Moreover, miR-10b and miR-196b have been related to angiogenesis and cancer metastasis [125–129] and are both upregulated in murine ECs treated with tumour-conditioned medium, although only miR-10 responded to increased VEGF levels. These miRNAs are preferentially expressed in the vasculature of more invasive human breast tumours and are upregulated by tumour-produced growth factors in human ECs [63]. Taken together, these studies establish miR-10, and perhaps miR-196b, as potential new targets for the selective modulation of angiogenesis [123].

Zhou et al. reported that miR-503 simultaneously downregulates* FGF2* and* VEGFA*. miR-503 expression is inhibited in hepatocarcinoma cells and primary tumours which may be due to an epigenetic mechanism; its overexpression reduces tumour angiogenesis* in vitro* and* in vivo*, and furthermore, its expression is downregulated by hypoxia via HIF1*α*, thus indicating an antiangiogenesis role in tumorigenesis [130]. Finally, other studies have indicated that miRNAs may function as tumour suppressors by targeting p70S6K1. Two independent studies, the first with miR-128 in glioma [131] and the second with miR-145 in colon cancer tissues [132], demonstrated that decreased p70S6K1 expression, mediated by these miRNAs, inhibits cell proliferation, tumour growth, and angiogenesis which is thought to be because HIF-1*α* and VEGF are both downstream to this molecule.

In conclusion, the key angiogenic factor VEGF appears to be regulated by several miRNAs including miR-191, miR-126, miR-155, miR-31, the miR-17-92 cluster, miR-10, miR196, and miR-1/206 (summarized in Figure 2 and Table 1); however, exhaustive studies on the implication of these miRNAs in therapeutic treatments are needed before these findings can be added to existing therapeutic anti-VEGF drugs.

### 4.5. miRNAs That Affect Other Pathways Implicated in Angiogenesis

Other angiogenesis-modulating miRNAs that do not affect any of the previously described targets include miR-132, miR-26a, and miR-130a (Figure 2 and Table 1), the latter of which inhibits the expression of two antiangiogenic genes:* GAX* (growth arrest homeobox) and* HOXA5 *(homeobox A5) [133] and is produced in increased amounts by hECs (human embryonic carcinoma cells) in culture. Similarly, miR-132 is also highly upregulated in a human vasculogenesis model as well as in human tumour endothelium [134]. Its ectopic expression* in vitro* enhances EC proliferation and tubulogenesis. MiR-132 expression in hECs repressed p120RasGAP (its predicted target) increasing RAS activity and thus promoting angiogenesis, which could explain why p120RasGAP is expressed in normal but not in tumour endothelium [134]. Further, supporting this, the same group showed that the addition of anti-miR-132 inhibited angiogenesis in wild-type mice but not in mice with an inducible* Rasa1* (encoding p120RasGAP) deletion; in another experiment, targeted delivery of anti-miR-132 nanoparticles to the vessels restored p120RasGAP expression in the tumour endothelium, suppressing angiogenesis and decreasing the tumour burden in an orthotopic xenograft mouse model of human breast carcinoma. It is therefore thought that miR-132 acts as an angiogenic switch by suppressing endothelial p120RasGAP expression, resulting in Ras activation and induction of neovascularization which is counteracted by anti-miR-132 [134]. Taken together, these observations indicate that miR-132 may play an important role in pathological neovascularization downstream of multiple triggers, including tumour-derived growth factors, viral infections, and inflammation.

Recent studies in human HCCs (hepatocellular carcinoma cells) have demonstrated that miR-26a is implicated in tumour angiogenesis [135, 136]. Ectopic expression of miR-26a reduces VEGFA levels in HepG2 (human hepatocellular liver carcinoma cell line) cells. Furthermore,* in silico* analysis indicates that* PIK3C2*α is a downstream miR-26a target gene, and inhibition studies suggest that miR-26a decreases VEGFA expression in HCCs via the PI3K/AKT/HIF-1*α*/VEGFA pathway. Finally, VEGFA levels inversely correlate with miR-26a levels in HCC tumours [135], and there is also a correlation between miR-26a downregulation and increased angiogenic potential in HCCs [136]. In addition, HGF (hepatocyte growth factor) has been identified as a miR-26a target, demonstrating its antiangiogenic function which is at least partially mediated by inhibiting cMet (HGF-hepatocyte growth factor receptor) and its downstream signalling pathway, thus reducing VEGFA expression in HCCs and decreasing VEGFR-2 signaling in ECs [136].

## 5. “Angiogenic” miRNAs in the Era of Personalised Medicine

There are different therapeutic strategies for inhibiting miRNAs* in vivo* that are currently being evaluated in preclinical models (reviewed in [137]). These strategies include the following.

*Antagomirs*. They are a class of chemically engineered oligonucleotides which are able to silence endogenous miRNAs. They are specifically designed, chemically modified, cholesterol-conjugated single-stranded RNA analogues which are complementary to miRNA targets [138–140]. 

*Locked Nucleic Acid- (LNA-) AntimiRs*. They are antisense RNA oligonucleotides in which the ribose moiety of an LNA nucleotide is modified to increase stability and specificity. LNA nucleotides can be mixed with DNA or RNA residues in the oligonucleotide depending on the user's requirements [141]. 

*MiR Sponge*. This is miRNA-inhibiting transgene which expresses an mRNA which contains multiple tandem binding sites for an endogenous miRNA which is thus able to stably interact with the corresponding miRNA and prevent its association with its endogenous targets [142]. 

*miR-Mask*. This is a single-stranded 2′-O-methyl-modified antisense oligonucleotide which is fully complementary to the predicted miRNA binding sites in the 3′-UTRs of target mRNAs. The miR-mask is therefore able to obscure the access of the miRNA to its binding sites on the target mRNA and so impairs its inhibitory function [143].

Alternatively, there are strategies intended to restore miRNA levels, such as miRNA mimics. It is based on the use of double-stranded synthetic oligonucleotides that mimic endogenous miRNAs and are processed into the cell when they are transfected [144]. The expression vectors of these miRNAs are constructed with promoters that can enable the expression of certain miRNAs in a tissue-/tumour-specific manner [145]. For instance, a liposome-formulated mimic of the tumour suppressor miR-34 named MRX34 (developed by Mirna Therapeutics) produced a complete tumour regression in orthotopic mouse models of hepatocellular carcinoma [146]. These results prompted the development of an ongoing phase I multicentre clinical trial (ClinicalTrials.gov identifier: NCT01829971) to evaluate the safety of MRX34 in patients with primary liver cancer or those with liver metastasis from other cancers.

In summary, although the pharmacological manipulation of miRNAs is still in its initial steps, there are scientific evidences demonstrating the potent modulatory impact of certain miRNAs on the angiogenic response.

## 6. Conclusions

miRNAs are implicated in most, if not all, signalling pathways and in many human diseases, including cancer. This review summarizes the role of miRNAs in the control of different pathways related to angiogenesis and, in particular, with the tumour neovascularization. To date there are groups of well characterized miRNAs implicated in regulating EC function and angiogenesis, making them attractive objectives in tumour angiogenesis. Hopefully, in a few years targeted use of specific miRNAs against angiogenic pathways in cancer will become a reality, allowing combining these with other types of antitumour strategies.

## Figures and Tables

**Table 1:** Angiogenic miRNAs related to cancer and their targets.

miRNA	Role	Function	Targets	Reference
Dicer	Function loss	Maturation of microRNAs	miRNAs	[56, 57] [58, 60, 61]

miR-126	Proangiogenic	Regulates the response of endothelial cells to VEGF	SPRED-1, PIK3R2, VECAM-1,	[64, 68, 71]

miR-221/222	Antiangiogenic and proangiogenic	Inhibitor of SCF	C-KIT, eNOS, p27	[73] [61] [74]

miR-17-92 cluster	Proangiogenic and antiangiogenic	Regulation of vascular integrity	THBS1, p21, S1P, JAK1 Flk-1 (VEGFR-2)	[83] [86] [88]

let-7f; miR-27b	Proangiogenic	↑EC-mediated angiogenesis	∗ND	[75]

miR-214	Antiangiogenic	Tubular sprouting	Quaking	[89]

miR-93	Proangiogenic	Enhanced endothelial cell activity	LATS2	[90]

miR-210	Proangiogenic	Endothelial cell migration and formation of capillaries	Ephrin-A3	[97]

miR-20b	Antiangiogenic	Decreases levels of HIF1A and VEGF	VEGF, HIF-1*α*	[102, 103]

miR-21	Antiangiogenic	Induction of tumour angiogenesis, confers resistance to hypoxia	PTEN, PDCD4, Sprouty1	[104] [105]

miR-200 family	Antiangiogenic	Epithelial-mesenchymal transition	ETS-1	[109]

miR-200c	Antiangiogenic	Epithelial-mesenchymal transition	VEGFR-2	[110]

miR-107	Antiangiogenic	Hypoxia signalling	HIF-1*β*	[111]

miR-519c	Proangiogenic	Depletion of tumour angiogenesis	HIF-1*α*	[112]

miR-424	Proangiogenic and antiangiogenic	Destabilization of the E3-ligase assembly, increasing HIF-1*α* levels	CUL-2 VEGF VEGFR-2 FGFR-1	[113] [117]

miR-15a	Antiangiogenic	Control of the cell cycle, apoptosis, proliferation, and angiogenesis	BCL-2 VEGF-A	[116]

miR-16	Antiangiogenic	Controls VEGF expression and induces cell apoptosis	VEGF VEGFR-2 FGFR-1 BCL-2 VEGF-A	[117] [116]

miR-378	Proangiogenic	Cell survival and tumour growth	SUFU and FUS-1	[114, 119]

miR-296	Proangiogenic	Promotes angiogenesis by increasing levels of proangiogenic growth factor receptors	HGS	[120]

miR-199a	Antiangiogenic	Suppresses tumour angiogenesis via the HIF-1*α*/VEGF pathway	HER3	[121]

miR-125b	Antiangiogenic	Suppresses tumour angiogenesis via the HIF-1*α*/VEGF pathway	HER2 HER3	[121]

miR-361-5p	Antiangiogenic	Cancer development and progression	VEGF A	[122]

miR-1/206	Antiangiogenic	Regulation of VegfA expression	VEGF A	[124]

miR-10b	Proangiogenic	Regulation of endothelial cell division and migration	HOXD10, FLT1	[123]

miR-196b	Proangiogenic	∗ND	∗ND	[63]

miR-503	Antiangiogenic	Overexpression reduces tumour angiogenesis	FGF2, VEGFA	[130]

miR-128	Antiangiogenic	Decreases cell proliferation, tumour growth, and angiogenesis	P70S6K1	[131]

mir-145	Antiangiogenic	Inhibition of tumour growth and angiogenesis	P70S6K1	[132]

miR-130a	Proangiogenic	Increases angiogenesis by targeting GAX and HOXA5 (antiangiogenic genes)	GAX, HOXA5	[133]

miR-132	Proangiogenic	Increases Ras activity	p120RasGAP	[134]

miR-26a	Antiangiogenic	Suppresses tumour growth and metastasis	PIK3C2*α* HGF	[135] [136]

*ND: not described.

## References

[1] Lee RC, Feinbaum RL, Ambros V (1993). The C. elegans heterochronic gene *lin-4* encodes small RNAs with antisense complementarity to *lin-14*. *Cell*.

[2] Esquela-Kerscher A, Slack FJ (2006). Oncomirs—microRNAs with a role in cancer. *Nature Reviews Cancer*.

[3] Garzon R, Fabbri M, Cimmino A, Calin GA, Croce CM (2006). MicroRNA expression and function in cancer. *Trends in Molecular Medicine*.

[4] Meltzer PS (2005). Cancer genomics: small RNAs with big impacts. *Nature*.

[5] Lu J, Getz G, Miska EA (2005). MicroRNA expression profiles classify human cancers. *Nature*.

[6] Calin GA, Sevignani C, Dumitru CD (2004). Human microRNA genes are frequently located at fragile sites and genomic regions involved in cancers. *Proceedings of the National Academy of Sciences of the United States of America*.

[7] Calin GA, Croce CM (2006). MicroRNA signatures in human cancers. *Nature Reviews Cancer*.

[8] Lagos-Quintana M, Rauhut R, Lendeckel W, Tuschl T (2001). Identification of novel genes coding for small expressed RNAs. *Science*.

[9] Lau NC, Lim LP, Weinstein EG, Bartel DP (2001). An abundant class of tiny RNAs with probable regulatory roles in Caenorhabditis elegans. *Science*.

[10] Lee RC, Ambros V (2001). An extensive class of small RNAs in *Caenorhabditis elegans*. *Science*.

[11] Bartel DP (2009). MicroRNAs: Target Recognition and Regulatory Functions. *Cell*.

[12] Ul Hussain M (2012). Micro-RNAs (miRNAs): genomic organisation, biogenesis and mode of action. *Cell and Tissue Research*.

[13] Lee Y, Kim M, Han J (2004). MicroRNA genes are transcribed by RNA polymerase II. *The EMBO Journal*.

[14] Denli AM, Tops BBJ, Plasterk RHA, Ketting RF, Hannon GJ (2004). Processing of primary microRNAs by the Microprocessor complex. *Nature*.

[15] Gregory RI, Yan K, Amuthan G (2004). The Microprocessor complex mediates the genesis of microRNAs. *Nature*.

[16] Han J, Lee Y, Yeom K, Kim Y, Jin H, Kim VN (2004). The Drosha-DGCR8 complex in primary microRNA processing. *Genes and Development*.

[17] Han J, Lee Y, Yeom KH (2006). Molecular basis for the recognition of primary microRNAs by the Drosha-DGCR8 complex. *Cell*.

[18] Yi R, Qin Y, Macara IG, Cullen BR (2003). Exportin-5 mediates the nuclear export of pre-microRNAs and short hairpin RNAs. *Genes & Development*.

[19] Lund E, Güttinger S, Calado A, Dahlberg JE, Kutay U (2004). Nuclear Export of MicroRNA Precursors. *Science*.

[20] Bohnsack MT, Czaplinski K, Görlich D (2004). Exportin 5 is a RanGTP-dependent dsRNA-binding protein that mediates nuclear export of pre-miRNAs. *RNA*.

[21] Bernstein E, Caudy AA, Hammond SM, Hannon GJ (2001). Role for a bidentate ribonuclease in the initiation step of RNA interference. *Nature*.

[22] Hutvágner G, McLachlan J, Pasquinelli AE, Bálint É, Tuschl T, Zamore PD (2001). A cellular function for the RNA-interference enzyme dicer in the maturation of the let-7 small temporal RNA. *Science*.

[23] Hutvágner G, Zamore PD (2002). A microRNA in a multiple-turnover RNAi enzyme complex. *Science*.

[24] Mourelatos Z, Dostie J, Paushkin S (2002). miRNPs: a novel class of ribonucleoproteins containing numerous microRNAs. *Genes and Development*.

[25] Carthew RW, Sontheimer EJ (2009). Origins and Mechanisms of miRNAs and siRNAs. *Cell*.

[26] Krol J, Loedige I, Filipowicz W (2010). The widespread regulation of microRNA biogenesis, function and decay. *Nature Reviews Genetics*.

[27] Farazi TA, Hoell JI, Morozov P, Tuschl T (2013). MicroRNAs in human cancer. *Advances in Experimental Medicine and Biology*.

[28] Calin GA, Dumitru CD, Shimizu M (2002). Frequent deletions and down-regulation of micro-RNA genes miR15 and miR16 at 13q14 in chronic lymphocytic leukemia. *Proceedings of the National Academy of Sciences of the United States of America*.

[29] Tagawa H, Seto M (2005). A microRNA cluster as a target of genomic amplification in malignant lymphoma. *Leukemia*.

[30] Huse JT, Brennan C, Hambardzumyan D (2009). The PTEN-regulating microRNA miR-26a is amplified in high-grade glioma and facilitates gliomagenesis in vivo. *Genes and Development*.

[31] Takamizawa J, Konishi H, Yanagisawa K (2004). Reduced expression of the let-7 microRNAs in human lung cancers in association with shortened postoperative survival. *Cancer Research*.

[32] Mayr C, Hemann MT, Bartel DP (2007). Disrupting the pairing between let-7 and Hmga2 enhances oncogenic transformation. *Science*.

[33] Chin LJ, Ratner E, Leng S (2008). A SNP in a let-7 microRNA complementary site in the KRAS 3′ untranslated region increases non-small cell lung cancer risk. *Cancer Research*.

[34] Jiang S, Zhang H, Lu M (2010). MicroRNA-155 functions as an oncomiR in breast cancer by targeting the suppressor of cytokine signaling 1 gene. *Cancer Research*.

[35] Han L, Witmer PD, Casey E, Valle D, Sukumar S (2007). DNA methylation regulates microRNA expression. *Cancer Biology & Therapy*.

[36] Saito Y, Jones PA (2006). Epigenetic activation of tumor suppressor microRNAs in human cancer cells. *Cell Cycle*.

[37] Saito Y, Liang G, Egger G (2006). Specific activation of microRNA-127 with downregulation of the proto-oncogene BCL6 by chromatin-modifying drugs in human cancer cells. *Cancer Cell*.

[38] Lehmann U, Hasemeier B, Christgen M (2008). Epigenetic inactivation of microRNA gene hsa-mir-9-1 in human breast cancer. *Journal of Pathology*.

[39] Chang T, Wentzel EA, Kent OA (2007). Transactivation of miR-34a by p53 Broadly Influences Gene Expression and Promotes Apoptosis. *Molecular Cell*.

[40] He L, He X, Lowe SW, Hannon GJ (2007). microRNAs join the p53 network—another piece in the tumour-suppression puzzle. *Nature Reviews Cancer*.

[41] Landgraf P, Rusu M, Sheridan R (2007). A mammalian microRNA expression atlas based on small RNA library sequencing. *Cell*.

[42] Hatley ME, Patrick DM, Garcia MR (2010). Modulation of K-Ras-dependent lung tumorigenesis by MicroRNA-21. *Cancer Cell*.

[43] Huang T, Wu F, Loeb GB (2009). Up-regulation of miR-21 by HER2/neu signaling promotes cell invasion. *Journal of Biological Chemistry*.

[44] O'Donnell KA, Wentzel EA, Zeller KI, Dang CV, Mendell JT (2005). c-Myc-regulated microRNAs modulate E2F1 expression. *Nature*.

[45] He L, Thomson JM, Hemann MT (2005). A microRNA polycistron as a potential human oncogene. *Nature*.

[46] Folkman J (2003). Fundamental concepts of the angiogenic process. *Current Molecular Medicine*.

[47] Veikkola T, Karkkainen M, Claesson-Welsh L, Alitalo K (2000). Regulation of angiogenesis via vascular endothelial growth factor receptors. *Cancer Research*.

[48] Zetter BR (1998). Angiogenesis and tumor metastasis. *Annual Review of Medicine*.

[49] Roskoski R (2007). Vascular endothelial growth factor (VEGF) signaling in tumor progression. *Critical Reviews in Oncology Hematology*.

[50] Gupta MK, Qin RY (2003). Mechanism and its regulation of tumor-induced angiogenesis. *World Journal of Gastroenterology*.

[51] Bergers G, Benjamin LE (2003). Tumorigenesis and the angiogenic switch. *Nature Reviews Cancer*.

[52] Papetti M, Herman IM (2002). Mechanisms of normal and tumor-derived angiogenesis. *The American Journal of Physiology—Cell Physiology*.

[53] Carmeliet P (2005). Angiogenesis in life, disease and medicine. *Nature*.

[54] Katoh M (2013). Therapeutics targeting angiogenesis: genetics and epigenetics, extracellular miRNAs and signaling networks (Review). *International Journal of Molecular Medicine*.

[55] Welti J, Loges S, Dimmeler S, Carmeliet P (2013). Recent molecular discoveries in angiogenesis and antiangiogenic therapies in cancer. *Journal of Clinical Investigation*.

[56] Bernstein E, Kim SY, Carmell MA (2003). Dicer is essential for mouse development. *Nature Genetics*.

[57] Wienholds E, Koudijs MJ, Van Eeden FJM, Cuppen E, Plasterk RHA (2003). The microRNA-producing enzyme Dicer1 is essential for zebrafish development. *Nature Genetics*.

[58] Yang WJ, Yang DD, Na S, Sandusky GE, Zhang Q, Zhao G (2005). Dicer is required for embryonic angiogenesis during mouse development. *The Journal of Biological Chemistry*.

[59] Giraldez AJ, Cinalli RM, Glasner ME (2005). MicroRNAs regulate brain morphogenesis in zebrafish. *Science*.

[60] Kuehbacher A, Urbich C, Zeiher AM, Dimmeler S (2007). Role of Dicer and Drosha for endothelial microRNA expression and angiogenesis. *Circulation Research*.

[61] Suárez Y, Fernández-Hernando C, Pober JS, Sessa WC (2007). Dicer dependent microRNAs regulate gene expression and functions in human endothelial cells. *Circulation Research*.

[62] Ruby JG, Jan CH, Bartel DP (2007). Intronic microRNA precursors that bypass Drosha processing. *Nature*.

[63] Plummer PN, Freeman R, Taft RJ (2013). MicroRNAs regulate tumor angiogenesis modulated by endothelial progenitor cells. *Cancer Research*.

[64] Fish JE, Santoro MM, Morton SU (2008). miR-126 regulates angiogenic signaling and vascular integrity. *Developmental Cell*.

[65] Parker LH, Schmidt M, Jin S (2004). The endothelial-cell-derived secreted factor Egfl7 regulates vascular tube formation. *Nature*.

[66] Campagnolo L, Leahy A, Chitnis S (2005). EGFL7 is a chemoattractant for endothelial cells and is up-regulated in angiogenesis and arterial injury. *The American Journal of Pathology*.

[67] Soncin F, Mattot V, Lionneton F (2003). VE-statin, an endothelial repressor of smooth muscle cell migration. *EMBO Journal*.

[68] Wang S, Aurora AB, Johnson BA (2008). The endothelial-specific microRNA miR-126 governs vascular integrity and angiogenesis. *Developmental Cell*.

[69] Lagos-Quintana M, Rauhut R, Yalcin A, Meyer J, Lendeckel W, Tuschl T (2002). Identification of tissue-specific MicroRNAs from mouse. *Current Biology*.

[70] Wienholds E, Kloosterman WP, Miska E (2005). MicroRNA expression in zebrafish embryonic development. *Science*.

[71] Kuhnert F, Mancuso MR, Hampton J (2008). Attribution of vascular phenotypes of the murine Egf17 locus to the microRNA miR-126. *Development*.

[72] Png KJ, Halberg N, Yoshida M, Tavazoie SF (2012). A microRNA regulon that mediates endothelial recruitment and metastasis by cancer cells. *Nature*.

[73] Poliseno L, Tuccoli A, Mariani L (2006). MicroRNAs modulate the angiogenic properties of HUVECs. *Blood*.

[74] Le Sage C, Nagel R, Egan DA (2007). Regulation of the p27Kip1 tumor suppressor by miR-221 and miR-222 promotes cancer cell proliferation. *The EMBO Journal*.

[75] Suárez Y, Sessa WC (2009). MicroRNAs as novel regulators of angiogenesis. *Circulation Research*.

[76] Papapetropoulos A, García-Cardeña G, Madri JA, Sessa WC (1997). Nitric oxide production contributes to the angiogenic properties of vascular endothelial growth factor in human endothelial cells. *Journal of Clinical Investigation*.

[77] Dentelli P, Rosso A, Orso F, Olgasi C, Taverna D, Brizzi MF (2010). MicroRNA-222 controls neovascularization by regulating signal transducer and activator of transcription 5A expression. *Arteriosclerosis, Thrombosis, and Vascular Biology*.

[78] Nicoli S, Knyphausen C, Zhu LJ, Lakshmanan A, Lawson ND (2012). MiR-221 is required for endothelial tip cell behaviors during vascular development. *Developmental Cell*.

[79] Pineau P, Volinia S, McJunkin K (2010). miR-221 overexpression contributes to liver tumorigenesis. *Proceedings of the National Academy of Sciences of the United States of America*.

[80] Venturini L, Battmer K, Castoldi M (2007). Expression of the miR-17-92 polycistron in chronic myeloid leukemia (CML) CD34+ cells. *Blood*.

[81] Mendell JT (2008). miRiad roles for the miR-17-92 cluster in development and disease. *Cell*.

[82] Ota A, Tagawa H, Karnan S (2004). Identification and characterization of a novel gene, C13orf25, as a target for 13q31-q32 amplification in malignant lymphoma. *Cancer Research*.

[83] Kuhnert F, Kuo CJ (2010). miR-17-92 angiogenesis micromanagement. *Blood*.

[84] Tréguer K, Heinrich E, Ohtani K, Bonauer A, Dimmeler S (2012). Role of the microRNA-17-92 cluster in the endothelial differentiation of stem cells. *Journal of Vascular Research*.

[85] Dews M, Homayouni A, Yu D (2006). Augmentation of tumor angiogenesis by a Myc-activated microRNA cluster. *Nature Genetics*.

[86] Doebele C, Bonauer A, Fischer A (2010). Members of the microRNA-17–92 cluster exhibit a cell-intrinsic antiangiogenic function in endothelial cells. *Blood*.

[87] Bonauer A, Carmona G, Iwasaki M (2009). MicroRNA-92a controls angiogenesis and functional recovery of ischemic tissues in Mice. *Science*.

[88] Yin R, Wang R, Guo L, Zhang W, Lu Y (2013). MiR-17-3p inhibits angiogenesis by downregulating flk-1 in the cell growth signal pathway. *Journal of Vascular Research*.

[89] van Mil A, Grundmann S, Goumans M (2012). MicroRNA-214 inhibits angiogenesis by targeting Quaking and reducing angiogenic growth factor release. *Cardiovascular Research*.

[90] Fang L, Du WW, Yang W (2012). MiR-93 enhances angiogenesis and metastasis by targeting LATS2. *Cell Cycle*.

[91] Huang X, Le Q-T, Giaccia AJ (2010). MiR-210—micromanager of the hypoxia pathway. *Trends in Molecular Medicine*.

[92] Brown JM, Giaccia AJ (1998). The unique physiology of solid tumors: Opportunities (and problems) for cancer therapy. *Cancer Research*.

[93] Carmeliet P, Jain RK (2000). Angiogenesis in cancer and other diseases. *Nature*.

[94] Liu Y, Cox SR, Morita T, Kourembanas S (1995). Hypoxia regulates vascular endothelial growth factor gene expression in endothelial cells: Identification of a 5′ enhancer. *Circulation Research*.

[95] Shweiki D, Itin A, Soffer D, Keshet E (1992). Vascular endothelial growth factor induced by hypoxia may mediate hypoxia-initiated angiogenesis. *Nature*.

[96] Foekens JA, Sieuwerts AM, Smid M (2008). Four miRNAs associated with aggressiveness of lymph node-negative, estrogen receptor-positive human breast cancer. *Proceedings of the National Academy of Sciences of the United States of America*.

[97] Fasanaro P, D'Alessandra Y, Di Stefano V (2008). MicroRNA-210 modulates endothelial cell response to hypoxia and inhibits the receptor tyrosine kinase ligand ephrin-A3. *Journal of Biological Chemistry*.

[98] Lou YL, Guo F, Liu FL (2012). MiR-210 activates notch signaling pathway in angiogenesis induced by cerebral ischemia. *Molecular and Cellular Biochemistry*.

[99] Kosaka N, Iguchi H, Hagiwara K, Yoshioka Y, Takeshita F, Ochiya T (2013). Neutral sphingomyelinase 2 (nSMase2)-dependent exosomal transfer of angiogenic micrornas regulate cancer cell metastasis. *The Journal of Biological Chemistry*.

[100] Ho AS, Huang X, Cao H (2010). Circulating miR-210 as a novel hypoxia marker in pancreatic cancer. *Translational Oncology*.

[101] Zhao A, Li G, Péoc'h M, Genin C, Gigante M (2013). Serum miR-210 as a novel biomarker for molecular diagnosis of clear cell renal cell carcinoma. *Experimental and Molecular Pathology*.

[102] Cascio S, D'Andrea A, Ferla R (2010). miR-20b modulates VEGF expression by targeting HIF-1α and STAT3 in MCF-7 breast cancer cells. *Journal of Cellular Physiology*.

[103] Lei Z, Li B, Yang Z (2009). Regulation of HIF-1α and VEGF by miR-20b tunes tumor cells to adapt to the alteration of oxygen concentration. *PLoS ONE*.

[104] Liu LZ, Li C, Chen Q (2011). Mir-21 induced angiogenesis through AKT and ERK activation and HIF-1α expression. *PLoS ONE*.

[105] Polytarchou C, Iliopoulos D, Hatziapostolou M (2011). Akt2 regulates all Akt isoforms and promotes resistance to hypoxia through induction of miR-21 upon oxygen deprivation. *Cancer Research*.

[106] Gregory PA, Bert AG, Paterson EL (2008). The miR-200 family and miR-205 regulate epithelial to mesenchymal transition by targeting ZEB1 and SIP1. *Nature Cell Biology*.

[107] Korpal M, Lee ES, Hu G, Kang Y (2008). The miR-200 family inhibits epithelial-mesenchymal transition and cancer cell migration by direct targeting of E-cadherin transcriptional repressors ZEB1 and ZEB2. *The Journal of Biological Chemistry*.

[108] Park S, Gaur AB, Lengyel E, Peter ME (2008). The miR-200 family determines the epithelial phenotype of cancer cells by targeting the E-cadherin repressors ZEB1 and ZEB2. *Genes and Development*.

[109] Chan YC, Khanna S, Roy S, Sen CK (2011). MiR-200b targets Ets-1 and is down-regulated by hypoxia to induce angiogenic response of endothelial cells. *The Journal of Biological Chemistry*.

[110] Shi L, Zhang S, Wu H (2013). MiR-200c increases the radiosensitivity of non-small-cell lung cancer cell line A549 by targeting VEGF-VEGFR2 pathway. *PLoS ONE*.

[111] Yamakuchi M, Lotterman CD, Bao C (2010). P53-induced microRNA-107 inhibits HIF-1 and tumor angiogenesis. *Proceedings of the National Academy of Sciences of the United States of America*.

[112] Cha ST, Chen PS, Johansson G (2010). MicroRNA-519c suppresses hypoxia-inducible factor-1α expression and tumor angiogenesis. *Cancer Research*.

[113] Ghosh G, Subramanian IV, Adhikari N (2010). Hypoxia-induced microRNA-424 expression in human endothelial cells regulates HIF-*α* isoforms and promotes angiogenesis. *The Journal of Clinical Investigation*.

[114] Hua Z, Lv Q, Ye W (2006). Mirna-directed regulation of VEGF and other angiogenic under hypoxia. *PLoS ONE*.

[115] Cimmino A, Calin GA, Fabbri M (2005). miR-15 and miR-16 induce apoptosis by targeting BCL2. *Proceedings of the National Academy of Sciences of the United States of America*.

[116] Sun CY, She XM, Qin YB (2013). miR-15a and miR-16 affect the angiogenesis of multiple myeloma by targeting VEGF. *Carcinogenesis*.

[117] Chamorro-Jorganes A, Araldi E, Penalva LOF, Sandhu D, Fernández-Hernando C, Suárez Y (2011). MicroRNA-16 and MicroRNA-424 regulate cell-autonomous angiogenic functions in endothelial cells via targeting vascular endothelial growth factor receptor-2 and fibroblast growth factor receptor-1. *Arteriosclerosis, Thrombosis, and Vascular Biology*.

[118] Dejean E, Renalier MH, Foisseau M (2011). Hypoxia-microRNA-16 downregulation induces VEGF expression in anaplastic lymphoma kinase (ALK)-positive anaplastic large-cell lymphomas. *Leukemia*.

[119] Lee DY, Deng Z, Wang CH, Yang BB (2007). MicroRNA-378 promotes cell survival, tumor growth, and angiogenesis by targeting SuFu and Fus-1 expression. *Proceedings of the National Academy of Sciences of the United States of America*.

[120] Würdinger T, Tannous BA, Saydam O (2008). miR-296 regulates growth factor receptor overexpression in angiogenic endothelial cells. *Cancer Cell*.

[121] He J, Jing Y, Li W (2013). Roles and Mechanism of miR-199a and miR-125b in Tumor Angiogenesis. *PLoS ONE*.

[122] Kanitz A, Imig J, Dziunycz PJ (2012). The expression levels of microRNA-361-5p and its target VEGFA are inversely correlated in human cutaneous squamous cell carcinoma. *PLoS ONE*.

[123] Hassel D, Cheng P, White MP (2012). MicroRNA-10 regulates the angiogenic behavior of zebrafish and human endothelial cells by promoting vascular endothelial growth factor signaling. *Circulation Research*.

[124] Stahlhut C, Suárez Y, Lu J, Mishima Y, Giraldez AJ (2012). miR-1 and miR-206 regulate angiogenesis by modulating VegfA expression in zebrafish. *Development*.

[125] Tehler D, Høyland-Kroghsbo NM, Lund AH (2011). The miR-10 microRNA precursor family. *RNA Biology*.

[126] Lund AH (2010). MiR-10 in development and cancer. *Cell Death and Differentiation*.

[127] Ma L, Teruya-Feldstein J, Weinberg RA (2007). Tumour invasion and metastasis initiated by microRNA-10b in breast cancer. *Nature*.

[128] O’Connell RM, Rao DS, Chaudhuri AA, Baltimore D (2010). Physiological and pathological roles for microRNAs in the immune system. *Nature Reviews Immunology*.

[129] Shen X, Fang J, Lv X (2011). Heparin impairs angiogenesis through inhibition of microRNA-10b. *The Journal of Biological Chemistry*.

[130] Zhou B, Ma R, Si W (2013). MicroRNA-503 targets FGF2 and VEGFA and inhibits tumor angiogenesis and growth. *Cancer Letters*.

[131] Shi Z-M, Wang J, Yan Z (2012). MiR-128 inhibits tumor growth and angiogenesis by targeting p70S6K1. *PLoS ONE*.

[132] Xu Q, Liu L, Qian X (2012). MiR-145 directly targets p70S6K1 in cancer cells to inhibit tumor growth and angiogenesis. *Nucleic Acids Research*.

[133] Chen Y, Gorski DH (2008). Regulation of angiogenesis through a microRNA (miR-130a) that down-regulates antiangiogenic homeobox genes *GAX* and *HOXA5*. *Blood*.

[134] Anand S, Majeti BK, Acevedo LM (2010). MicroRNA-132-mediated loss of p120RasGAP activates the endothelium to facilitate pathological angiogenesis. *Nature Medicine*.

[135] Chai ZT, Kong J, Zhu XD, Zhang YY, Lu L, Zhou JM (2013). MicroRNA-26a inhibits angiogenesis by down-regulating VEGFA through the PIK3C2alpha/Akt/HIF-1alpha pathway in hepatocellular carcinoma. *PLoS ONE*.

[136] Yang X, Zhang XF, Lu X (2014). MicroRNA-26a suppresses angiogenesis in human hepatocellular carcinoma by targeting hepatocyte growth factor-cMet pathway. *Hepatology*.

[137] Anand S (2013). A brief primer on microRNAs and their roles in angiogenesis. *Vascular Cell*.

[138] Esau C, Kang X, Peralta E (2004). MicroRNA-143 regulates adipocyte differentiation. *The Journal of Biological Chemistry*.

[139] Krützfeldt J, Rajewsky N, Braich R (2005). Silencing of microRNAs in vivo with ‘antagomirs’. *Nature*.

[140] Krützfeldt J, Kuwajima S, Braich R (2007). Specificity, duplex degradation and subcellular localization of antagomirs. *Nucleic Acids Research*.

[141] Elmén J, Lindow M, Silahtaroglu A (2008). Antagonism of microRNA-122 in mice by systemically administered LNA-antimiR leads to up-regulation of a large set of predicted target mRNAs in the liver. *Nucleic Acids Research*.

[142] Ebert MS, Sharp PA (2010). MicroRNA sponges: progress and possibilities. *RNA*.

[143] Esau C, Davis S, Murray SF (2006). miR-122 regulation of lipid metabolism revealed by in vivo antisense targeting. *Cell Metabolism*.

[144] Bader AG, Brown D, Stoudemire J, Lammers P (2011). Developing therapeutic microRNAs for cancer. *Gene Therapy*.

[145] Ji J, Shi J, Budhu A (2009). MicroRNA expression, survival, and response to interferon in liver cancer. *The New England Journal of Medicine*.

[146] Bader AG (2012). MiR-34 - a microRNA replacement therapy is headed to the clinic. *Frontiers in Genetics*.

